# Effect of Stacked Insecticidal Cry Proteins from Maize Pollen on Nurse Bees (*Apis mellifera carnica*) and Their Gut Bacteria

**DOI:** 10.1371/journal.pone.0059589

**Published:** 2013-03-22

**Authors:** Harmen P. Hendriksma, Meike Küting, Stephan Härtel, Astrid Näther, Anja B. Dohrmann, Ingolf Steffan-Dewenter, Christoph C. Tebbe

**Affiliations:** 1 Department of Animal Ecology and Tropical Biology, Biocentre, University of Würzburg, Würzburg, Germany; 2 Thünen Institute of Biodiversity, Federal Research Institute for Rural Areas, Forestry and Fisheries, Braunschweig, Germany; International Atomic Energy Agency, Austria

## Abstract

Honey bee pollination is a key ecosystem service to nature and agriculture. However, biosafety research on genetically modified crops rarely considers effects on nurse bees from intact colonies, even though they receive and primarily process the largest amount of pollen. The objective of this study was to analyze the response of nurse bees and their gut bacteria to pollen from Bt maize expressing three different insecticidal Cry proteins (Cry1A.105, Cry2Ab2, and Cry3Bb1). Naturally Cry proteins are produced by bacteria (*Bacillus thuringiensis*). Colonies of *Apis mellifera carnica* were kept during anthesis in flight cages on field plots with the Bt maize, two different conventionally bred maize varieties, and without cages, 1-km outside of the experimental maize field to allow *ad libitum* foraging to mixed pollen sources. During their 10-days life span, the consumption of Bt maize pollen had no effect on their survival rate, body weight and rates of pollen digestion compared to the conventional maize varieties. As indicated by ELISA-quantification of Cry1A.105 and Cry3Bb1, more than 98% of the recombinant proteins were degraded. Bacterial population sizes in the gut were not affected by the genetic modification. Bt-maize, conventional varieties and mixed pollen sources selected for significantly different bacterial communities which were, however, composed of the same dominant members, including *Proteobacteria* in the midgut and *Lactobacillus* sp. and *Bifidobacterium* sp. in the hindgut. Surprisingly, Cry proteins from natural sources, most likely *B. thuringiensis*, were detected in bees with no exposure to Bt maize. The natural occurrence of Cry proteins and the lack of detectable effects on nurse bees and their gut bacteria give no indication for harmful effects of this Bt maize on nurse honey bees.

## Introduction

During the last 15 years several regions of the world have explored the increasing introduction of transgenic crops into agriculture [1] and a significant proportion of them have been engineered to produce insecticidal proteins which are naturally synthesized by bacteria summarized under the species name *Bacillus thuringiensis* [2], [3]. Members of this species are considered to inhabit soil but they are also found in other environmental niches including phylloplane [4] and insects [5]. Their crystal delta-endotoxins (Cry proteins) are highly specific for certain groups of insects, and the recombination and expression of their encoding genes in transgenic crops (frequently named “Bt crops”), including maize (“Bt maize”), has conferred protection against important pests, i.e., Cry1Ab for the European corn borer (*Ostrinia nubilalis*; Lepidoptera) or Cry3Bb1 for the Western corn rootworm (*Diabrotica virgifera*; Coleoptera). Stacked Bt maize events, in which several different Cry proteins are expressed, have more recently been developed to provide simultaneous resistance towards several pests.

The safe use of stacked Bt maize in agriculture requires their environmental risk assessment, in which unintended adverse effects on non-target organisms expected to share the same ecosystem are analyzed. Cry proteins develop their toxicity by forming pores in the gut epithelium of their target insects as a consequence of binding to specific receptors in the epithelial membrane [6]. While single Cry proteins have extensively been assessed for adverse side-effects on non-target organisms, the combination of several may result in additive or synergistic effects, because different Cry proteins may share binding sites [2]. Therefore stacked events may require a specific risk assessment beyond an evaluation of their single transformation events [7], [8].

Due to their high ecological and economic importance as pollinators and producers of honey, honey bees (*Apis mellifera*) are considered a focal non-target insect in environmental risk assessments of genetically modified crops [9], [10]. In most ecosystems, honey bees have access to a number of different pollen sources within their foraging range [11], but in agricultural landscapes with large-scale monocultures, pollen foragers may be forced to almost exclusively collect pollen from a single source, even from wind pollinated crops like maize [12]. A number of studies on effects of purified or pollen-enclosed single Cry proteins demonstrate that there is to date no indication of acute or chronic toxicity either for larvae or adult bees [13]–[16]. However, there is a lack of information whether this insecticidal specificity for functional bee colonies is maintained in crops expressing several different Cry proteins.

Within a honey bee colony, the exposure to Cry protein-containing pollen is different depending on the bees’ life-stage. The highest exposure can be expected for nurse bees because of their central function to convert bee bread (fermented pollen collected by forager bees) into dietary proteins which they then pass on to the bee brood [17], [18]. During this life-stage, which lasts from day 3 to day 11 after hatching, pollen is accumulated inside their gut [19]. Because of their central role in food supply, even sublethal negative effects of Cry proteins on nurse bees could thus have far reaching consequences for colony fitness.

Sublethal effects on honey bees triggered by consumption of Cry protein containing pollen have been studied by analyzing their physiological characteristics (e.g., weight of their body or their hypopharyngal gland) [20], [21] or behavior (e.g., foraging activity, learning performance) [21], [22]. Furthermore, it has been suggested that the intestinal bacterial community could be a sensitive indicator for an altered intestinal physiology [23]. The gut bacterial community of insects is considered to be important for nutrient acquisition and pathogen defense [24](Vasquez et al., 2012, PLoS ONE) and, in honey bees, the bacterial community structure is highly conserved [25], [26]. For the colony collapse disorder, a threat to *A. mellifera* populations, alterations in the bacterial community structure have been reported [27]. While laboratory studies with Cry1Ab supplemented pollen did not reveal significant alterations of the gut bacterial community structure of adult honey bees [23] there is no information whether this also holds true for nurse bees exposed to pollen with stacked Cry proteins under field conditions. Interestingly, bacteria of the genus *Bacillus* have frequently been isolated from gut material of bees, but among those, the Cry protein producing *B. thuringiensis* has not been detected [28], [29]. This suggests that bees might not be naturally adapted to Cry proteins as they would encounter them in Bt maize fields during anthesis.

The objective of this study was to analyze whether the presence of stacked Cry proteins in maize pollen would affect nurse bees and their gut bacteria in bee colonies exposed to Bt maize during anthesis. To provide an extreme but not unrealistic scenario of exposure, colonies of *A*. *mellifera carnica* were kept in cages within replicated field plots with Bt maize. The Bt maize selected for this study was MON89034 × MON88017, a hybrid expressing three Cry proteins (Cry1A.105, Cry2Ab2, Cry3Bb1) in their pollen. This maize variety is already grown in different parts of the world and used for food and feed [30], [31] but their specific effect on nurse bees has not been analyzed. As controls, bees were kept under the same conditions in plots with two conventional varieties. Furthermore, additional controls of nurse bees from colonies without cage and *ad libitum* access to mixed pollen sources were also considered. Maize pollen digestibility and the Cry protein concentrations in the gut of nurse bees were analyzed. It was also analyzed whether Cry proteins from other sources (native *B. thuringiensis*) could occur in the gut of bees not exposed to Bt maize. Consequences of the different pollen diets, including those with stacked Cry proteins for the gut bacterial community were analyzed from directly extracted DNA of gut material by PCR-based cultivation-independent quantification, fingerprinting and DNA-sequencing of the bacterial 16S rRNA genes.

## Materials and Methods

### Experimental Field Setup

A 6-ha experimental maize field at the Thünen-Institute consisted of 40 randomized plots (30 m×42 m) of which 24 were used in this study (see Figure S1). These plots were part of a randomized plot design and represented three different maize varieties (“treatments”). The genetically modified Bt maize was the hybrid MON 89034 × MON 88017 (indicated here as “treatment” BT) in the genetic background of the conventional variety DKC 5143. The other two maize varieties were DKC 5143 with no genetic modification (treatment DKC) and Benicia (BEN). The maize varieties were sown on May 18^th^ 2009. Seeds were obtained from Monsanto (Düsseldorf, Germany) and Pioneer HiBreed (Buxtehude, Germany). The Bt maize produces three different insecticidal delta-endotoxins: Cry1A.105, Cry2Ab2, Cry3Bb1, and the enzyme EPSPS (5-enolpyruvylshikimate-3-phosphate synthase). Cry1A.105 is a chimeric protein comprising domains of Cry1Ab, Cry1F and Cry1Ac [32]. All delta-endotoxins of this study are naturally produced by strains of *Bacillus thuringiensis* subspecies *kumamotoensis*. The gene encoding for the EPSPS originates from *Agrobacterium* sp. CP4 and confers tolerance towards the herbicidal compound glyphosate. In 2009, the expression levels in maize pollen of this study were 4.2 µg g^−1^ (fresh weight) for Cry1A.105, 1.2 µg g^−1^ for Cry2Ab2, 7.0 µg g^−1^ for Cry3Bb1 and 170 µg/g for CP4-EPSPS [16]). No Cry proteins were detected in material from the conventional maize varieties. Calculations of exposure levels to honey bees in this study refer to 1-µg average fresh weight of one pollen grain.

Five days before the onset of anthesis (August 1^st^, BEN; August 8^th^, BT and DKC), artificial swarms of *Apis mellifera carnica* were prepared from one breeding line (Institute for Apiculture Celle). Each new colony contained one queen with approximately 1,100 workers (122.9 g bee biomass ±7.2 SD, n = 49 colonies). All queens were sisters mated with a controlled drone population. The polystyrene hives (24 cm×15 cm×17 cm, Apidea™ Vertriebs AG, Steinhausen, Switzerland) had three empty frames (10 cm×10 cm) to build combs and, the bees were given *ad libitum* access to a 72% invert sugar (glucose, fructose) solution (Apiinvert, Südzucker AG, Mannheim, Germany).

The placement of the standardized honey bee colonies to the maize pollen was synchronized to anthesis of the different maize varieties. As soon as 5 to 10% of the maize anthers had opened, two colonies were put into a flight cage within the experimental plots (see Figure S1). Each field plot of this study contained one flight cage. One cage covered a 48 m^2^ area with a height of 3 m, with the gauze having a 1.3 mm mesh-width. Simultaneously to the placement of colonies in the flight cages, 8 honey bee colonies with *ad libitum* access of various pollens sources were placed without cages in a field with flowering *Phacelia tanacetifolia* (treatment PHA) at 1 km distance from this experimental field site.

Synchronized to the peak anthesis time of the individual maize varieties, freshly hatched worker bees (<24 h) from the *Apis mellifera carnica* donor colonies were marked with a pen and added to the experimental colonies (mean 23 bees per colony; a total of 1130 bees). At the dates of field sampling, the colonies contained newly built wax combs with maize pollen and sugar stores and open brood. The marked bees were recollected after 9 d of in-hive exposure, thus sampling 10-d old nurse bees. Bees were frozen at −70°C.

In addition, 24 bees from the *A. mellifera carnica* donor-colonies were collected and immediately frozen at −70°C as controls. They originated from colonies 50 km north of the experimental field site, at the Institute of Apiculture (Celle) sampled on August 1^st^, August 8^th^ and September 28^th^; 3 times eight bees). It should be noted that these bees did not come in contact with commercial Bt products, as neither Bt plants are admitted to grow in Germany, nor Bt based anti-waxmoth treatments were used at the apiary.

### Analyses of Nurse Bees

To monitor for lethal and/or sublethal effects, the rate of retrieval and the weight of the bees (including intestine) were analyzed. To isolate the gut material, all nurse bees were dissected immediately after thawing. The midgut and hindgut were separately transferred to sterile 1.5 ml polypropylene tubes and kept on ice. A total of 300 µL sterile PBST buffer (*137 mM NaCl, 27 mM KCl, 100 mM Na_2_HPO_4_, 10 mM KH_2_PO_4_, 0.5% Tween-20, pH 7.4*) was added. The gut material was manually stirred with a sterile pipette tip, followed by 20 s of vortexing. For each gut segment, a 50 µL sample volume was stored for pollen analyses (−20°C). The remaining suspension was centrifuged at 16,200×*g* and 4°C for 10 min and the supernatant was analyzed for Cry proteins. Before extraction of bacterial DNA, the centrifuged pellets were stored at −70°C.

The survival rates of the test bees were determined by the proportion between retrieved and non-retrieved bees of the marked cohort of introduced nurses. Bees from a total of 49 colonies (14 from BT, 14 from DKC, 13 from BEN and 8 from PHA) were examined. The survival rates of the different colonies were analyzed with a generalized linear mixed model with the logit function and a binomial error distribution. The weight of the nurse bees was measured at the moment of their retrieval (n = 195 for BT; 201 for DKC; 219 for BEN; 99 for PHA).

The weighted average pollen digestion within bees was analyzed, using four 0.9 µLL replicate gut samples per bee (See Table S1). The pollen grains were counted using microscopic examination at 100×magnification in a counting chamber (Neubauer improved haemocytometer; Carl Roth, Karlsruhe, Germany). An absence of other pollen than maize pollen indicated that the experimental colonies and nurse bees were not contaminated with external pollen sources.

The level of digestion was scored according to three classes: not digested (0–20%), partly digested (20–80%) or totally digested (80–100%) [33], [34]). The mean digestion rate per class 10%, 50% and 90%) were used to calculate a weighted digestion rate per bee, as based on the relative abundance of pollen per class. Bee-weight and pollen-digestion data were analyzed on the colony level with a linear mixed effects model. The three models (survival, weight, digestion) all included the treatment (BT, DKC, BEN, PHA) as a fixed effect, and colony pairs within the same cage as a random effect and the colony background of bees as a nested random effect [35]. The models were fitted using the package ‘lme4’ in R [36], [37], and the results were reported significant at p-values <0.05.

### Quantification of Cry Proteins

For the quantification of Cry proteins, 100 µL of the supernatants obtained from the gut content in PBST buffer were subjected to ELISA (enzyme-linked immunosorbent assay, supplied by Monsanto), targeting the Cry proteins Cry1A.105 and Cry3Bb1, respectively. No test was available in this study for Cry2Ab2. The antibody reaction products were quantified at 450 nm wavelength. The detection limit (DTC) was determined for each ELISA test plate [38]. The average DTC for Cry1A.105 was 0.56 ng mL^−1^, corresponding to 0.17 ng Cry1A.105 per bee gut. For Cry3Bb1 it was 0.40 ng mL^−1^, corresponding to 0.12 ng Cry3Bb1 per bee gut. A one-way analysis of variance (ANOVA) was used to evaluate the differences in Cry protein contents of different gut samples higher than the DTC implementing the Holm-Sidak method for pair wise multiple comparisons (SigmaPlot, Systat Software, Erkrath, Germany). The correlation of the number of pollen and the concentration of Cry1A.105 and Cry3Bb1 was established in analyses of 32 individual nurse bees (4 replicates originating from 8 different hives from 6 cages of 5 different plots; due to a quantitative analytical constraint limited in sampling size) and determined by a linear regression analysis in SigmaPlot (Systat Software). All data values below the DTC were omitted in the regression analyses. The bias, by excluding low concentration values did not alter the positive nature of the correlation, as verified by substituting all non-detect values by zeros [39]. Normal distribution of data analyzed with Shapiro-Wilk.

### Detection of Native Cry Proteins from *Bacillus thuringiensis*

Serial dilutions of four *B. thuringiensis* strains were analyzed to test the response of the ELISA system to natural Cry proteins. The bacterial strains were obtained from the DSMZ (Leibniz Institute, Braunschweig, Germany). *B. thuringiensis* ssp. *kurstaki*, strain HD-1 (DSM 6102) and HD-73 (DSM 6101), produce Cry1Aa1, Cry1Ab3, Cry1Ab4, Cry1Ab10, Cry1Ac13, Cry1Ia3, Cry2Aa2, Cry2Ab1, Cry2Ab2 or Cry1Ac1, Cry1Ac7, Cry1Ac8 respectively [3]. *B. thuringiensis* ssp. *aizawai* strain HD-11 (DSM 6099) and HD-282 (DSM 6100) also produce Cry proteins (no detailed information was available). *Bacillus subtilis* 168 (DSM 402) was used as a negative control. All strains were cultivated aerobically at 28°C in liquid nutrient medium supplemented with MnSO_4_ for better sporulation (5 g L^−1^ peptone, 3 g L^−1^ meat extract, 60 µM MnSO_4_, pH 7.0). Growth of the cultures was followed by microscopic counts of cells using a Thoma counting chamber (Carl Roth). After 5 d of cultivation at 28°C, the liquid cultures were shifted to 4°C without shaking for sporulation. The sporulation efficiency was almost 100%.

### DNA Extraction and Microbial Community Analysis

The frozen pellets containing the gut material were thawed by adding 650 µL sterile PBST buffer. The suspensions were stirred and after centrifugation for 10 min at 100×*g* DNA was extracted from the supernatants using the FastDNA SPIN kit for soil and a FastPrep-24 system (both from MP Biomedicals, Eschwege, Germany) for bead beating. DNA was photometrically quantified with the NanoDrop 2000c (Thermo Fisher Scientific, Epsom, United Kingdom). A 100 µL-DNA solution from midgut contained approx. 8 ng µL^−1^ and hindgut 9.4 ng µL^−1^. The DNA solutions were stored at 4°C.

### Analyses of the Bacterial Abundance, Diversity and Community Similarities

For each treatment, 24 replicate nurse bees (4 replicates from 6 hives) were analysed of their hind- and midgut contents. The abundance of the bacterial 16S rRNA genes was determined by a quantitative real-time PCR (qPCR) applying universal bacterial primers F27 and Eub338rev [40] and the Maxima SYBR green/fluorescence qPCR Master Mix (Thermo Fisher Scientific Fermentas, Waltham, MA). The performance of the qPCR system was evaluated according to Bustin *et al.* [41]. The efficiency was 85.9% and all C_t_-values were within the linear range of the standards.

Terminal restriction fragment length polymorphism (T-RFLP) was applied to estimate the relative abundance and phylotype richness (diversity) of the dominant bacterial community members. T-RFLP profiles were conducted as described elsewhere [23] but were run on a CEQ8800™ Genetic Analysis System (Beckman Coulter, Krefeld, Germany), requiring a Cy5-labeled forward primer 27F and an unlabeled reverse primer 1378R. Terminal restriction fragments (T-RFs) representing less than 1.5% of the total peak heights were considered as background noise and excluded. Rare peaks which occurred in less than 3% of all profiles (less than 5 of 192 profiles) were not considered. T-tests were applied for the identification of significant differences in the abundance of particular T-RFs from different treatments. Significant differences were only considered to be indicative for a respective treatment, if the T-RF occurred in more than 80% of the replicates.

Comparisons of T-RFLP-profiles were carried out with PAST (PAleontological STatistics, version 1.79; http://folk.uio.no/ohammer/past; [42]. Bray-Curtis index [43] was used to generate similarity matrices and analysis of similarities (ANOSIM) was performed [44]. ANOSIM compares the ranks of distances between groups with ranks of distances within groups. In the resulting R-test statistic, high values (R>0.75) are commonly interpreted as “well separated”, medium values (0.75>R>0.25) as “separated but overlapping” and low values (R<0.25) as “barely separable” [45]. Diversity patterns of gut bacterial communities were visualized by non-metric multidimensional scaling (NMDS) ordination approach. The importance of particular environmental variables, i.e., treatments (BT, DKC, BEN, PHA), pollen numbers, bacterial 16S rRNA gene copy numbers and Cry protein concentrations, were analyzed by redundancy analysis (RDA) using R [36].

### DNA-sequencing of Bacterial 16S rRNA Genes and Phylogenetic Analyses

The 16S rRNA genes were PCR-amplified with unlabelled primers 27F and 1378R and the PCR products were cloned in *E. coli* JM109. In order to compare the T-RFs of the cloned sequences to the theoretical fragment sizes obtained by *in silico* analyzes, all PCR products were sequenced in forward orientation. DNA sequencing was performed by GATC Biotech (Konstanz, Germany) and the sequences were processed by the MEGA4 software [46], analyzed using the BLASTN routine (www.ncbi.nlm.nih.gov/BLAST) and, for chimera check, with the Pintail tool (www.bioinformatics-toolkit.org). The taxonomic position was evaluated using the RDP classifier [47] and ARB [48]. All new sequences of this study are deposited in the EMBL Nucleotide Sequence Database (Accession numbers HE613272 to HE613312).

## Results

### Effects of the Pollen Diets on Bee Survival, Body Weight, and the Efficiency to Digest Maize Pollen

**Figure 1:**
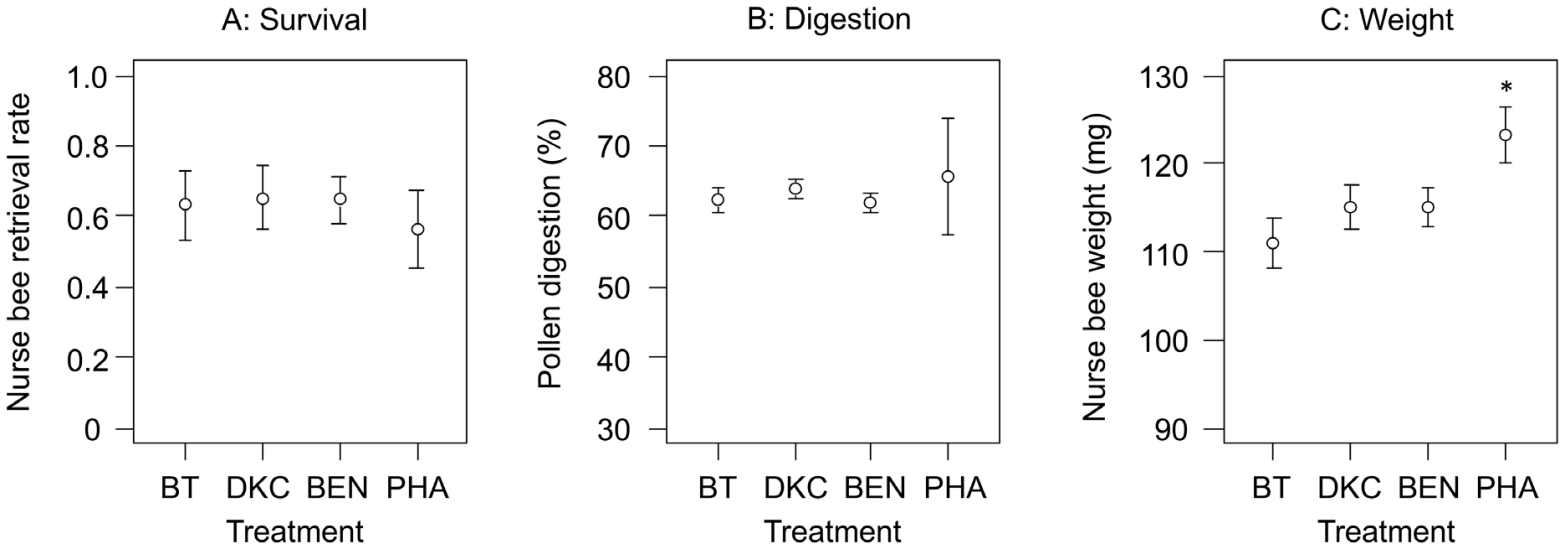
Response of nurse bees after a 9 d exposure period either to Bt maize (treatment BT), or two conventional maize cultivars (DKC, BEN), or controls with *ad libitum* access to different pollen sources from colonies kept at a Phacelia field (PHA). The survival (A) was indicated by the retrieval rate of marked bees, their weight (B) was determined at the moment of their retrieval. Microscopic analysis of bee hindguts was performed to calculate a weighted average degree of maize pollen digestion (C). The error bars indicate 95% confidence intervals. *indicates significant difference of a specific treatment.

From a total of 1,130 introduced bees, 714 bees were retrieved after 9 d, corresponding to 63% survival. On the basis of 373 microscopically examined nurse bees, a total of 41,703 pollen grains were rated for their level of digestion. Only 1,131 grains were found undigested, leaving more than 97% of the pollen partly or fully digested (partially 26,146; fully: 14,408 grains). Bt maize pollen, compared to the other maize pollen treatments (DKC, BEN, PHA), did not affect survival rates (Fig. 1A; *Chisq = *0.95, *Df* = 3, *P* = 0.81) or the weighted average pollen digestion rates of nurse bees (Fig. 1B; *F*_(3,18) = _2.29, *P* = 0.11), with the overall weighted digestion rate at 62.7%. The control colonies (PHA), with *ad libitum* access to Phacelia and other pollen sources, had significantly heavier nurse bees than the maize treatments (Fig. 1C; *F*_(3,18) = _4.61, *P* = 0.015). Between the different maize treatments BT, DKC and BEN in flight cages, no difference in body weights were found (Fig. 1C; F_(2,18) = _1.12, *P* = 0.34).

The amount of maize pollen found in the hindgut of the nurse bees kept in field plots with Bt maize was on average 16,000 pollen grains, though the variability between individual bees was high, with a standard deviation of 85.7% (Table S1). Prevalence of Bt-maize pollen was found restricted to the hindgut of the respective bees. The maize pollen uptake in colonies with free flying bees (PHA) was less frequent (528 grains, with a standard deviation of 71%). These pollen grains did not necessarily originate from maize plants grown at the experimental site (distance 1000 m), as other maize fields were located in closer vicinity (>250 m).

### Detection of Cry Proteins from the Bee Gut

**Figure 2:**
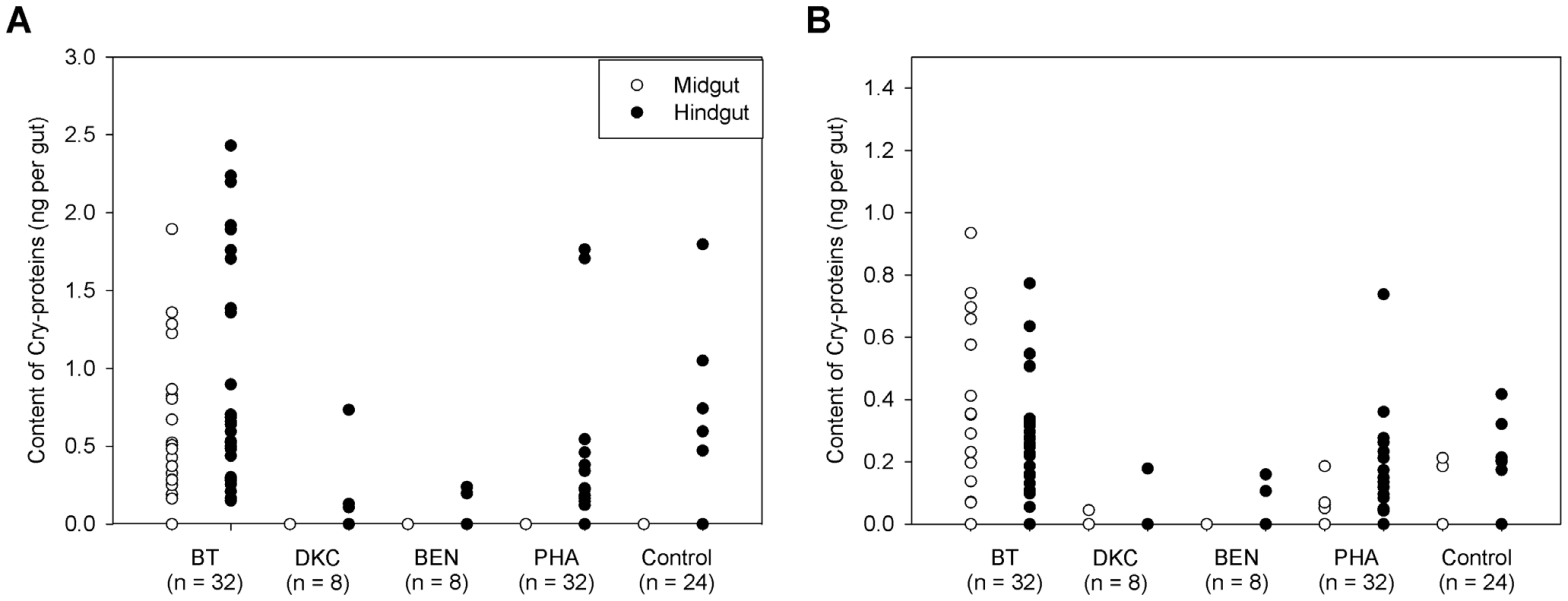
Quantification of Cry1A.105 (A) and Cry3Bb1 (B) from mid- and hindgut samples of nurse bees exposed to Bt maize (treatment BT), other conventionally bred maize varieties (DKC, BEN) or other pollen sources (Phacelia, control); n indicates the numbers of replicate samples analyzed. Each individual sample is represented by a circle. Samples below the detection limit were set to zero.

Nurse bees were analyzed for presence of Cry1A.105 and Cry3Bb1 in their mid- and hindgut (Fig. 2). For the bees from the Bt maize plots, 100% of the analyzed hindgut samples were positive for Cry1A.105 and 81% for Cry3Bb1 (Cry1A.105 0.91±0.69 ng (positive n = 32) and Cry3Bb1 0.29±0.17 ng (positive n = 26)). The detection of Cry proteins in the midgut was less frequent: 66% were positive for Cry1A.105 and 50% for Cry3Bb1. In cases of positive detection, the respective amounts of the Cry proteins were not significantly different in the mid- and hindgut, even though pollen numbers of the hindgut clearly exceeded those of the midgut. However, while the amounts of Cry1A.105 and Cry3Bb1 were comparable in the midgut, significantly more Cry1A.105 compared to Cry3Bb1 was detected in the hindgut, suggesting higher instability of Cry3Bb1 after passage through the gut.

**Figure 3:**
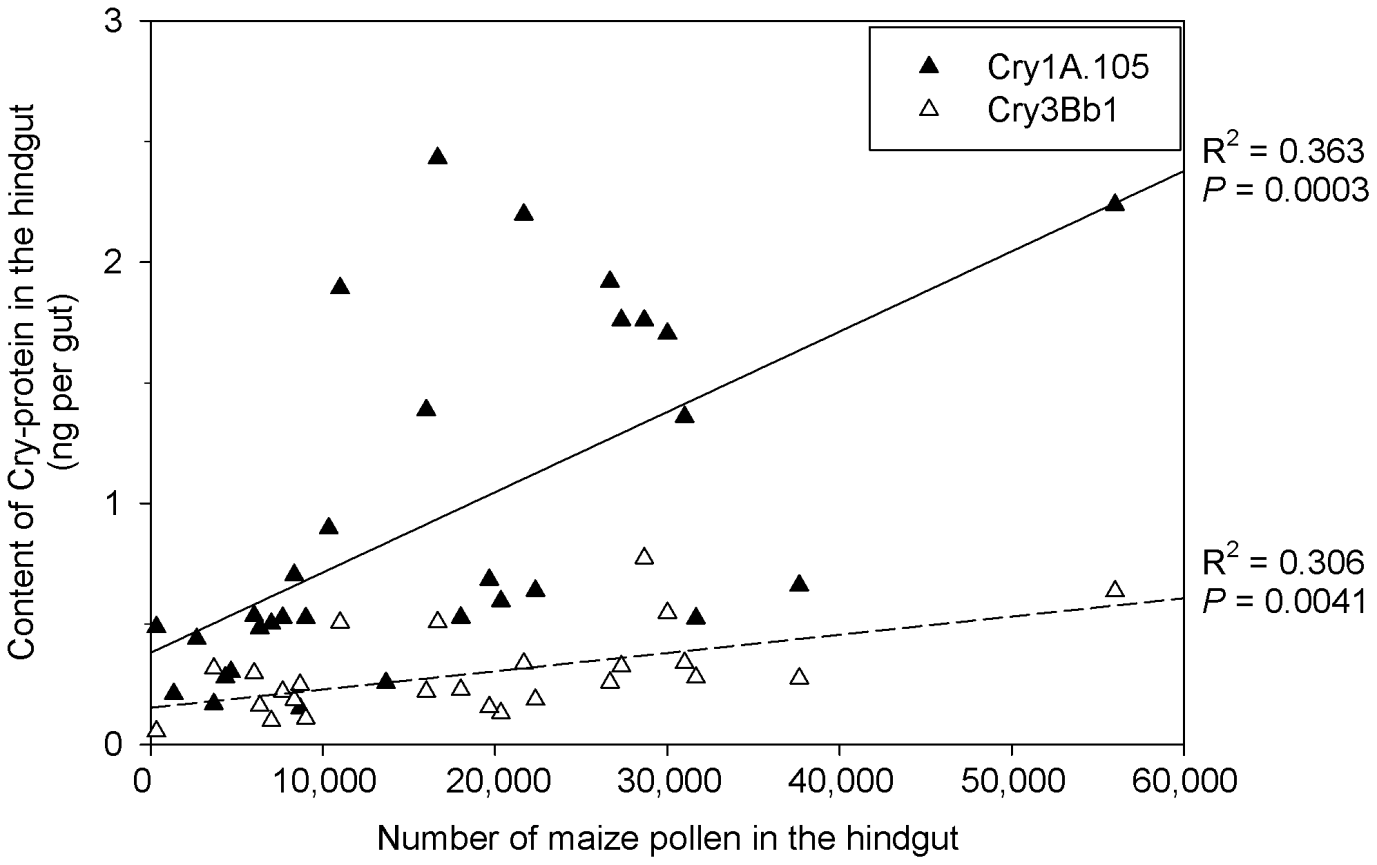
Correlation between the contents of Cry proteins for Cry1A.105 and Cry3Bb1, and maize pollen detected in the hindgut of nurse bees from colonies caged in field plots with Bt maize MON 89034 × MON 88017 during anthesis. Correlation data in the graph excluded values below the detection limit (DTC).

The relatively high variability of the Cry protein concentrations (standard deviation of 76% for Cry1A.105; 59% for Cry3Bb1) in the hindgut of the nurse bees from the plots with Bt maize was linked to the different amounts of pollen ingested by the individual bees, as underlined by the positive correlation of both Cry1A.105 and Cry3Bb1 with the respective pollen numbers in their hindguts (Fig. 3). Considering the concentrations of Cry proteins of intact pollen (see Materials and Methods), the ingestion of 16,000 Bt pollen grains per bee, digested by 61.7% (see Fig. 1B), would have resulted in a release of 42 ng Cry1A.105 and 69 ng Cry3Bb1 into the gut lumen. However, the actual amounts detected were much smaller, with 0.80±0.62 ng for Cry1A.105 and 0.33±0.21 ng for Cry3Bb1 in mid- and hindgut together, indicating degradation rates of 98.1% for Cry1A.105 and 99.5% of the Cry3Bb1, respectively.

Remarkably, Cry proteins were also detected, even though less frequently, but with similar concentrations in nurse bees with no direct exposure to Bt maize, i.e., from colonies of the DKC, BEN and PHA treatments and even in controls for which exposure to recombinant Cry proteins could be excluded (Fig. 2). In contrast to the BT treatment, Cry protein detection from the other treatments and controls was mainly in hindgut samples, which were positive for one or both Cry proteins in 38% of the nurse bees kept in the maize field plots and in 60% of those from colonies with the free flying bees (PHA). This result from the PHA treatment was especially remarkable since in 68% of the positive samples no maize pollen grains were detected, and for those, which contained maize pollen, there was no correlation between the concentrations of the Cry1A.105 and Cry3Bb1 proteins. This was also the case for the unexposed controls.

### Origin of Cry Proteins in Bees

For the control group of nurse bees with no exposure to Bt maize, five of 24 midgut samples were positive for Cry1A.105, and six for Cry3Bb1. Notably, two Cry3Bb1 positive midgut samples occurred in absence of a parallel Cry1A.105 detection in mid- or hindgut. This presence of Cry3Bb1 in absence of Cry1A.105 was never seen with nurse bees from the Bt treatment (where the digestion of Bt pollen would release both Cry1A.105 and Cry3Bb1 proteins), suggesting that Cry proteins detected in the control group originated from other, natural sources, i.e., bacteria belonging to *B. thuringiensis*.

Since the synthetic Cry1A.105 protein, for which the ELISA applied in this study had been developed, could not occur in natural *B. thuringiensis* strains, the positive signals, suggesting amounts between 0.5 to 1.8 ng “Cry1A.105” in their hindgut, must have been caused by cross-reaction with natural Cry1A or other proteins. Not all extracts of the bee guts responded in the ELISA, which excluded false-positive detection by other gut-derived proteins. Furthermore, additional pure culture studies with sporulated *B. thuringiensis* strains demonstrated that cross-reaction with the Cry1A.105 antibody in fact occurred, while controls with *Bacillus subtilis* were negative (Figure S2). The correlation between sporulated cell numbers and Cry1A.105 signal intensities allowed the calculation of *B. thuringiensis* spore/cell numbers which would be required for detection of natural Cry1A-like proteins with the Cry1A.105-specific ELISA (Table 1). Only 50 sporulated cells of *B. thuringiensis* ssp. *kurstaki* HD-73 were sufficient to cause an ELISA signal equivalent to 1 ng Cry1A.105 in a bees gut. In comparison 914 cells of another *kurstaki* strain were required for the same response. In contrast, more than 10^7^ spore/cell numbers were necessary for indicating 1 ng with two *B. thuringiensis* ssp. *aizawai s*trains, suggesting that either expression of Cry1A-proteins was low or antibodies were not specific for their particular Cry proteins.

**Table 1:** Hypothetical numbers of sporulated cells of *B. thuringiensis* strains expressing natural Cry proteins required for the detection of a Cry1A.105 equivalent by ELISA.

*B. thuringiensis* strain	Number of cells necessary in a bees gut samples to give an ELISA above DTC^a^
ssp. *kurstaki* HD-73 (DSM 6101)	2.50×10^1^±1.20×10^1^
ssp. *kurstaki* HD-1 (DSM 6102)	9.14×10^2^±5.86×10^2^
ssp. *aizawai* HD-11 (DSM 6099)	3.26×10^7^±0.14×10^7^
ssp. *aizawai* HD-282 (DSM 6100)	1.30×10^8^±0.98×10^8^

a refers to an extraction volume of 300 µL; DTC, detection threshold was 0.5 mg mL^−1.^

### Bacterial Diversity in Response to Pollen Exposure

**Figure 4:**
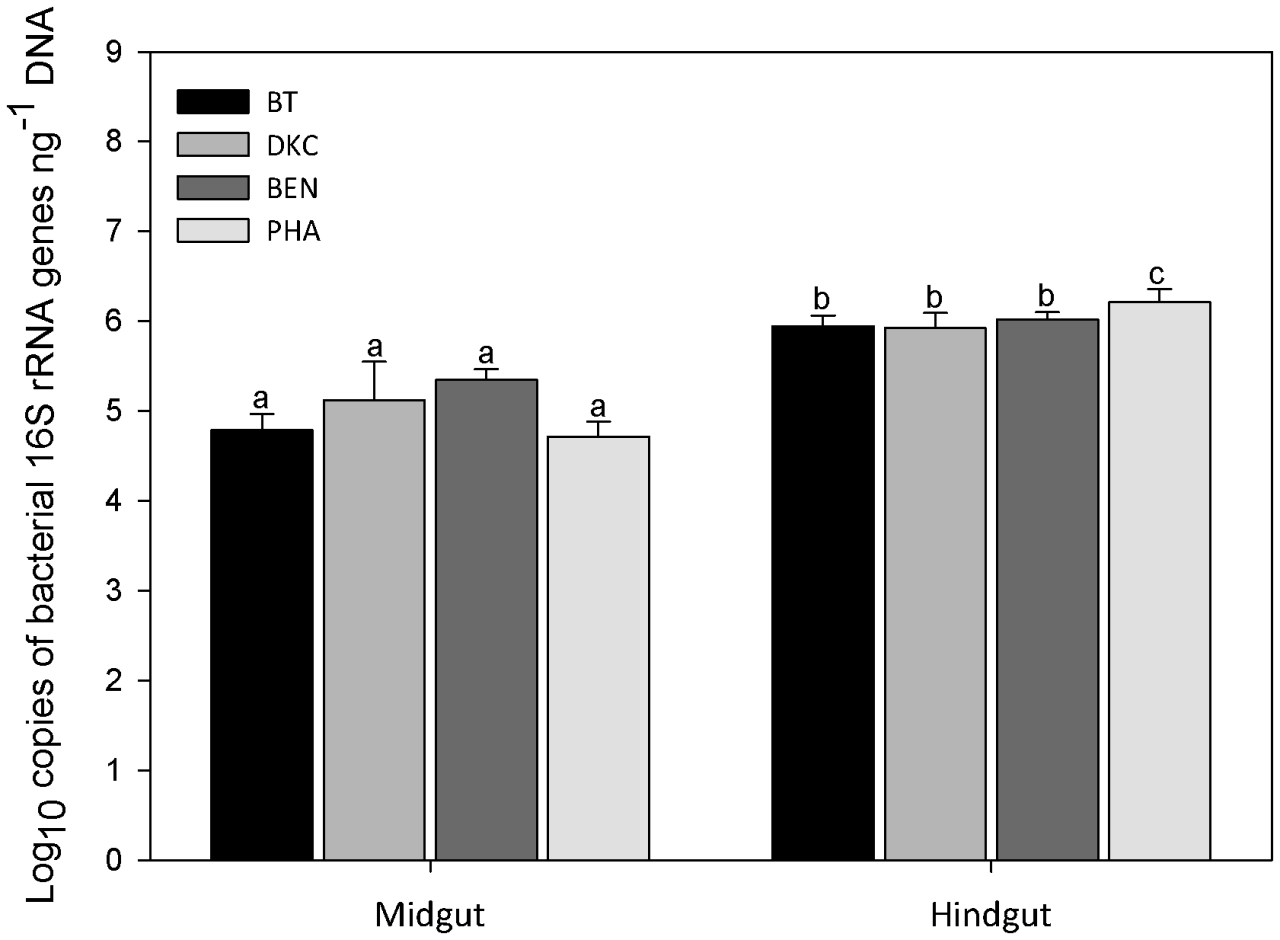
Copy numbers of bacterial 16S rRNA genes in gut material from nurse bees as determined by qPCR. Different letters on top of columns indicate significant differences. Nurse bees originated from colonies exposed to Bt maize MON 89034 × MON 88017 (treatment BT), conventional maize varieties (DKC, BEN) or other pollen sources including Phacelia (PHA).

The different maize pollen diets from BT, DKC and BEN had no significant effect on the overall bacterial abundance in midgut or hindgut (Fig. 4). In contrast, the bacterial population sizes in the hindgut, but not midgut, from nurse bees from the free-flying colonies (PHA) were significantly higher.

Assuming an average bacterial genome size of 5 Mbp and four 16S rRNA gene operons, the expected maximal copy number amplifiable from one ng total DNA would correspond to 7×10^5^. Copy numbers detected in the hindgut ranged from 2×10^5^ to 3×10^6^ (Fig. 4) and thus it appeared that the majority of DNA extracted from the hindgut was in fact of bacterial and not of pollen origin. With a total rRNA gene copy number of 3×10^6^ rRNA per ng DNA, 100 µL of hindgut DNA with a 9.4 ng DNA µL^−1^ would indicate under these assumptions a bacterial population size of 7×10^8^ cells in the hindgut.

**Figure 5:**
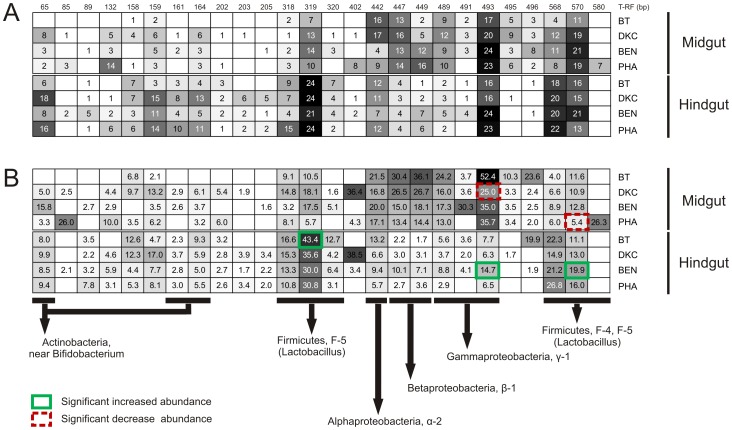
Schematic figure on the incidence (A) and abundance (B) of bacterial phylotypes detected by T-RFLP based on 16S rRNA genes. The T-RF patterns for each treatment, i.e., exposure to Bt maize (BT), two conventional maize varieties (DKC, BEN) and mixed pollen sources including Phacelia (PHA), are based on 24 replicates from individual bees. Frequencies of incidences and abundances are indicated by squares and correlate with the grey scale. Abundance values in **B** indicate % of a particular T-RF in relation to total TRFs of the corresponding TRFP-profiles. Abundance values were averaged only from scored T-RFs. Significant differences in abundances of frequently occurring T-RFs are indicated with coloured boarder lines. Bacterial phylotypes indicated by the particular T-RFs were identified by DNA-sequencing (see also Table S2).

Richness of bacterial phylotypes was determined by T-RFLP and profiles of individual bees revealed 1 to 11 T-RFs for their midgut (average 5.6±2.5), and 3 to 11 (6.8±1.6) for their hindgut. Based on DNA sequencing the consistently occurring T-RFs could be assigned to different taxa (Fig. 5; Table S2). The profiles of the midgut were mainly composed of *Proteobacteria*, while those of the hindgut were dominated by *Lactobacillus* (*Firmicutes*) and *Bifidobacterium* (*Actinobacteria*). ANOSIM confirmed significant differences between the diversity of bacteria from midgut and hindgut (R = 0.538, *P<*0.001).

All of the identified T-RFs in this study showed their highest similarities (97–99%) to bacterial 16S rRNA gene DNA sequences previously found in bees (mainly *Apis mellifera*) [25] (Table S2). With the exception of *Bartonella* sp. α-1 and *Proteobacterium* γ-2, all phylotypes proposed as consistent inhabitants of the bee gut [23], [25], were detected independent of the pollen source (treatment). The highest incidence of bacterial phylotypes was found for *Lactobacillus* F-5 in the hindgut (97%; n = 96) (Fig. 5 A) which was significantly more abundant in BT (Fig. 5 B). Two frequently occurring T-RFs (493, indicating a γ-1 *Proteobacterium* and 570 indicating a *Lactobacillus* F-4 or F-5) from hindgut were significantly higher with BEN. There was no indication for presence of indigenous *B. thuringiensis* (hypothetical T-RF 147 bp).

**Figure 6:**
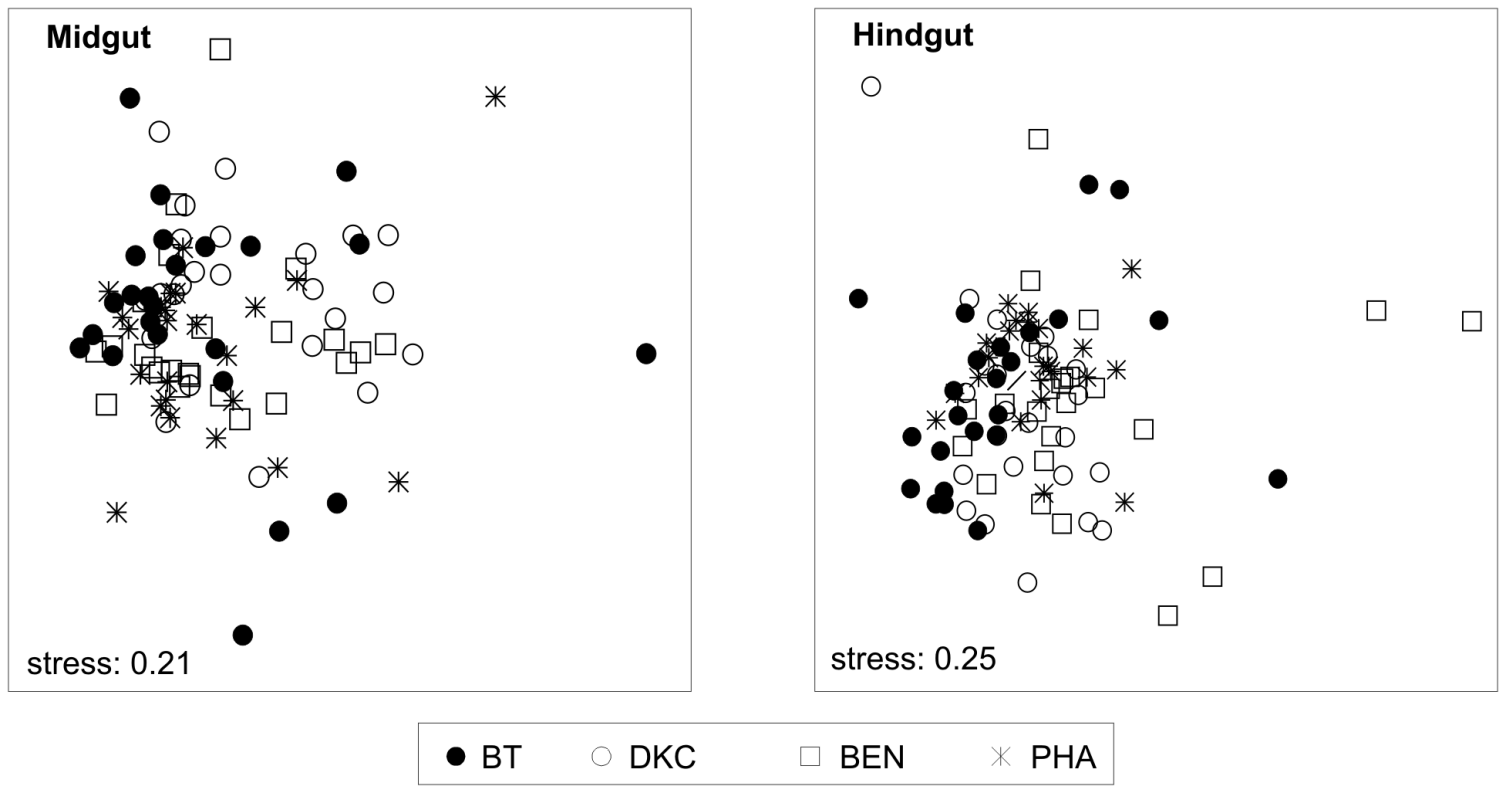
Nonmetric multidimensional scaling (NMDS) ordination plot of β-diversity patterns of bacterial community differences represented as Bray–Curtis distances of T-RFLP profiles. Stress values (0.21, 0.25) indicate that the distance between points in the ordination plot is a good representation of the degree of similarity between the bacterial communities in each sample. Each point represents the gut bacterial community obtained an individual nurse bee. Treatments: BT, exposure to pollen of Bt maize, DKC and BEN to conventional maize and PHA to other pollen sources including Phacelia.

Nonmetric multidimensional scaling (NMDS) visualized that the overall bacterial community structure in midgut and hindgut was not clearly affected by the particular pollen source (Fig. 6). For each treatment, analysis of similarities (ANOSIM) however indicated significant differences, even though the profiles were barely separable (midgut, R = 0.083; hindgut, R = 0.123) (Table S3). Similarly, for any comparisons between two treatments, ANOSIM revealed significant differences but only barely separable profiles for both midgut and hindgut, with the exception of BT and its near isogenic DKC from the midgut, which were not different. RDA revealed that for midgut 6.6%, and for hindgut 10.2% of the variability of the variance of the community profiles could be explained by the treatment. The content of Cry1A.105 and Cry3Bb1 had a relatively low explanatory value, explaining 1.5% and 4.8% of the variance of community profiles from the midguts and 2.0% and 1.6% from the hindguts. In the hindgut, the number of maize pollen explained 3.4%, whereas the bacterial community abundance (copy numbers of bacterial 16S rRNA genes) accounted for 2.4% and 3.6% in mid- and hindgut, respectively. Overall, the selected environmental variables explained 15.3% of the variability in the bacterial community structures of the midgut and 20.8% of the hindgut.

## Discussion

The flight cages used in this study forced the bee colonies to cover their protein demand exclusively from pollen of a particular maize variety (treatments BT, DKC, BEN). Interestingly, the gut of nurse bees from colonies of the free-flying foragers (treatment PHA) also contained some maize pollen, indicating that bees actively forage on pollen of this wind pollinated crop even with abundant access to alternative pollen sources [12].

Upon ingestion of maize pollen by nurse bees, only 3% remained undigested, which confirmed digestion rates found with other Bt maize [34]. This indicates that in fact Cry proteins from Bt maize pollen are released at large amounts into the gut lumen of the bees. In contrast to fully grown worker bee larvae which contained approx. 2,000 pollen grains [34], the gut of the nurse bees in this study contained on average eight times more, confirming the underlying assumption of this study that exposure of Cry proteins from Bt maize pollen in nurses is relatively high.

Once released into the gut, the insecticidal proteins may potentially interact with resident bacteria and the gut epithelium. However, the detected concentrations of Cry1A.105 and Cry3Bb1 indicated that the majority of the Cry proteins, i.e., more than 98%, were degraded. The fate of the third Cry protein, Cry2Ab2, was not analyzed and additional data on the persistence of this protein would be desirable. However, proteolysis is common in the bee gut and important for the acquisition of nutrients [19], [49], suggesting that Cry proteins generally do not resist such digestive processes.

There was no indication from mortality or body weight data that the Bt maize pollen or their included Cry proteins exhibited any negative effect on the nurse bees. This confirms data from laboratory feeding studies on the lack of adverse effects of Cry protein containing pollen on individual bees outside of their social context [8], [14], [16], [20], [50]. No effect on bee colonies was found with Bt maize expressing another *cry1A* gene [21]. The amounts of Cry proteins which were released into the gut lumen of the nurse bees and the lack of effects on survival and body weight clearly demonstrate high tolerance towards these three insecticidal proteins. Target lepidoptera and their relatives already responded to less than 100 pollen with Cry1Ab in their diet [51], [52] while the presence of 16,000 pollen with the three different Cry proteins in the hindgut of nurse bees in this study had no apparent effect.

The bacterial diversity in the gut of the nurse bees was analyzed by PCR amplification of their 16S rRNA genes from DNA directly extracted from gut material of the bees. This approach yields genetic signatures (phylotypes) and avoids non-detection of bacteria which would fail to grow on laboratory media [53]. A large discrepancy between the bacterial community recovered by cultivation and independent of cultivation was found when the bacterial community of the same gut material from bees was analyzed with both methods [29], [54]. The dominant bacterial phylotypes detected in this study confirmed for the highly conserved bacterial community structure seen generally for adult bees in other studies applying a similar methodological approach [23], [25], [27], however, with the exception of *Bartonella* sp. α-1 and *Proteobacterium* γ-2. Genetic profiling of the dominant 16S rRNA genes by T-RFLP analyses on honey bees from Thailand also failed to detect the *Bartonella* sp. α-1 [55] possibly because their abundance was too small to be detected by T-RFLP [56].

Despite the highly conservative bacterial community structure ANOSIM revealed significant diet dependent differences, suggestion quantitative responses to particular properties of the respective diets. The high number of replicates (24 individual profiles for each treatment) analyzed in this study allowed to test the diet-dependent significance for specific TR-Fs (indicating phylotypes) and there was in fact a significantly higher abundance of a *Lactobacillus* from the F-5 group with Bt maize in the hindgut. However, significant differences were not typical for Bt maize but occurred also with other treatments. T-RFs of the *Proteobacterium* γ-1 or *Lactobacillus* F-4 and F-5 were more abundant in the hindgut of nurse bees feeding on Benicia (BEN) than on the other pollen sources. Differences between BT and conventional maize pollen sources were in the same range, suggesting that Cry proteins did not differ in their effects from other protein sources.

Multivariate statistical analyses (NMDS, RDA) also visualized that the differences to BT were in the same range as to other pollen sources. RDA indicated that only 7% and 10% of the variance of the gut bacterial community structure in midgut and hindgut were linked to the different pollen sources. Interestingly, the bacterial community structure selected by mixed pollen sources (treatment PHA) was not more distantly related to the ones exclusively receiving maize pollen. This may be explained by the fact that in this study the exposure of nurse bees and their gut bacteria was restricted to only nine days, i.e., coinciding with the nurse bee life-stage period and that at the onset of the incubation, all bees came from the same source as newborns. Thus, the conclusions on the lack of GMO-specific effect of the gut bacterial community in this study relate to immediate responses of their structural diversity to the different pollen sources.

The frequent detection of Cry proteins in bees from the donor colonies which had never been exposed to Bt maize or any other Bt crop was an unexpected result. There was no indication for presence of *B. thuringiensis*, which belongs to the *Bacillus cereus* group and is not distinguishable from *B. cereus* itself by their 16S rRNA genes [57], [58]. In accordance with this study, cultivation independent analyses have never indicated the presence of *B. cereus* among the dominant gut bacteria. On the other hand, *B. cereus* has been detected in the gut of bees by means of cultivation [59]. The detection of Cry proteins from gut material of *A. mellifera* in this study clearly indicates the presence of *B. thuringiensis* (the only producer of Cry proteins) as an inhabitant of the gut, even though, on a theoretical basis, it cannot be excluded that Cry proteins would also be produced by other yet unknown bacteria. Since the Cry protein producing bacteria obviously do not cause negative effects for *A. mellifera*, they may use this host for non-pathogenic rather than infective growth, as suggested for members of the *B. cereus* group, including *B. thuringiensis* [5]. As little as 50 spores of a *B. thuringiensis kurstaki* strain were sufficient to give rise to positive signals with the ELISA system applied why they were not picked up by the TRFLP analysis which only visualizes the dominant gut residing bacteria.

### Conclusion

This study shows that honey bee nurses which were forced to cover their full protein demand by pollen from a stacked Bt maize showed no apparent effects on survival rates, body weight and pollen digestibility. The community structure of the gut bacteria significantly responded to the different pollen diets, but differences found with the Bt maize pollen were in the range of those occurring between pollen from conventionally bred varieties or mixed pollen sources. The relatively low Cry protein concentration measurements compared to the high exposure of nurse bees indicate that the recombinant proteins were actively digested. The natural occurrence of Cry proteins in the gut of nurse bees with no exposure to Bt maize and the lack of detectable effects on nurse bees and their gut bacteria give no indication for harmful effects of this Bt maize on honey nurse bees.

## Supporting Information

Figure S1 Experimental field design (schematic overview). The figure illustrates the location of field plots on the 6-ha maize field site of this study. The “x” marks indicate the position of flight cages within the particular plots. Maize varieties grown in the plots are indicated by BT for Bt maize (Cry1A.105; Cry2Ab2, and Cry3Bb1 in the genetic background of DKC 5143), DKC, for the non-engineered near isogenic cultivar DKC 5143, and BEN for, the conventionally bred cultivar “Benicia”. Underlined names indicate plots from which nurse bees for analyzed for their intestinal Cry-proteins and bacterial community. For plot size and more details see Materials and methods. Empty squares without further indication represent maize field plots with other cultivars or treatments with no relevance for this study. At the onset of maize flowering, two honey bee colonies were introduced per flight cage. Note that an additional group of eight honey bee colonies, without being caged, was placed in 1 km distance to this site, with *ad libitum* access to pollen at a field with *Phacelia tanacetifolia*.
(TIF)

Figure S2 Quantification of natural Cry-protein expressed by four different *Bacillus thuringiensis* strains. The expression levels of natural Cry protein by four *B. thuringiensis* strains were detected with an ELISA targeting the synthetic protein Cry1A.105 as used in this study to detect the recombinant synthetic Cry1A.105 protein from Bt maize MON 89034 × MON 88017. The 12 data points are the highest diluted cell suspension with a signal above the respective detection limits. The results show for two type culture strains of ssp. kurstaki that a relatively low number of bacterial cells (spores) can result in detecting relative high amounts of Cry-protein. Contrastingly, the presence of a relative high numbers of the ssp. aizawai, show for two type culture strains, can result in detecting only low amounts of Cry-protein. No detection signal above the DTC was recorded for *Bacillus subtilis* 168 (DSM 402) (negative control). These results illustrate with the example of *B. thuringiensis* spp. *kurstaki* that Cry protein within the bee gut may originate from the presence of only a few bacterial cells (or spores).
(TIF)

Table S1 Consumption of Bt maize pollen by nurse bees within honey bee colonies. To indicate maize pollen exposure to bees, the pollen amount in midgut and hindgut samples was quantified by microscopic examination (Leitz Laboralux K, Wetzlar, Germany). By transferring each sample homogenate onto a counting device, complete pollen grains and fragments larger than half of a pollen grain were counted at 100×magnification within a 0.9 µL volume, at an 1∶4 dilution (Neubauer Improved haemocytometer, Laboroptik GmbH, Bad Homburg, Germany). Each count with four subsamples attributed with a factor 333 to the total number of pollen in the gut segment (0.9 µL/300 µL total sample volume). The counted pollen in the rectum samples (1305) indicated the presence of a total of 434,565 Bt-maize pollen; with an average exposure of 15,520 Bt-pollen per bee (n = 28), ±85.7% SD. Midgut samples did not contribute to additional exposure data of the Bt-maize pollen, because no pollen was observed (64 negative counts, in a total of 16 bees). The experimental colonies were free of pollen stores, and the nurse bees were at time of introduction less than 24 hrs old. As a result, no other pollen than maize pollen were found in the nurse bees from the bee cages.
(DOCX)

Table S2 Comparison of 16S rRNA gene sequences retrieved in this study from the gut material of Apis mellifera and their affiliation to known bacteria taxa and previously detected bacterial 16S rRNA gene signatures in other studies. For the corresponding terminal restriction fragment (TRF) sizes, please see also Fig. 5.
(DOCX)

Table S3 Comparison of bacterial community composition (T-RFLP profiles) between treatments (pollen source, i.e. BT, DKC, BEN, PHA) for mid- and hindgut by one-way analysis of similarities (ANOSIM) with Bray-Curtis similarity.
(DOCX)
