# Supplementary figures and images for: Effect of Stacked Insecticidal Cry Proteins from Maize Pollen on Nurse Bees (Apis mellifera carnica) and Their Gut Bacteria
Source: PLoS One. 2013 Mar 22;8(3):e59589. doi: 10.1371/journal.pone.0059589 (PMC3606186; doi:10.1371/journal.pone.0059589)

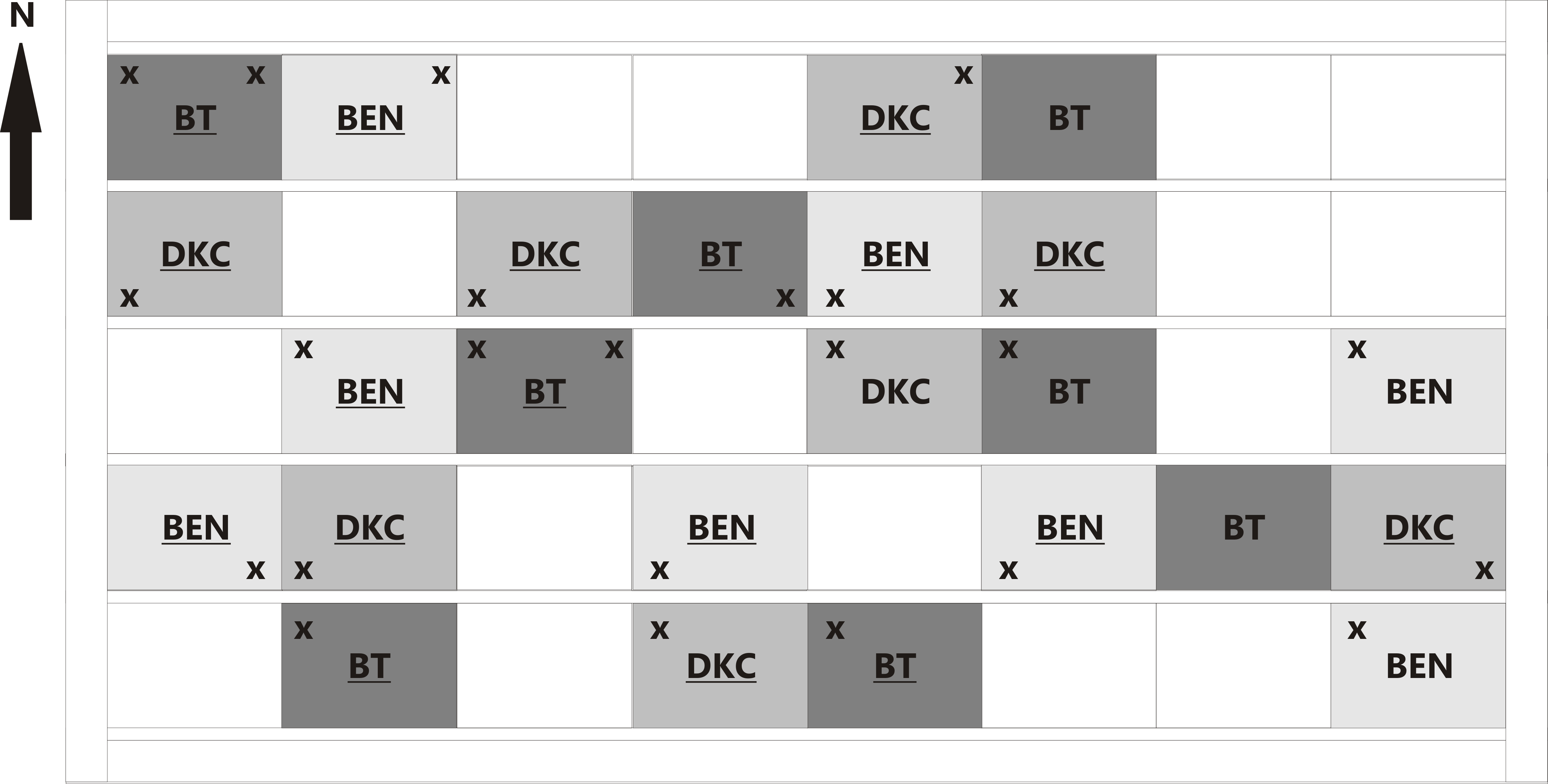

Supplement: Figure S1 — Experimental field design (schematic overview). The figure illustrates the location of field plots on the 6-ha maize field site of this study. The “x” marks indicate the position of flight cages within the particular plots. Maize varieties grown in the plots are indicated by BT for Bt maize (Cry1A.105; Cry2Ab2, and Cry3Bb1 in the genetic background of DKC 5143), DKC, for the non-engineered near isogenic cultivar DKC 5143, and BEN for, the conventionally bred cultivar “Benicia”. Underlined names indicate plots from which nurse bees for analyzed for their intestinal Cry-proteins and bacterial community. For plot size and more details see Materials and methods. Empty squares without further indication represent maize field plots with other cultivars or treatments with no relevance for this study. At the onset of maize flowering, two honey bee colonies were introduced per flight cage. Note that an additional group of eight honey bee colonies, without being caged, was placed in 1 km distance to this site, with ad libitum access to pollen at a field with Phacelia tanacetifolia. (TIF) [file pone.0059589.s001.tif]

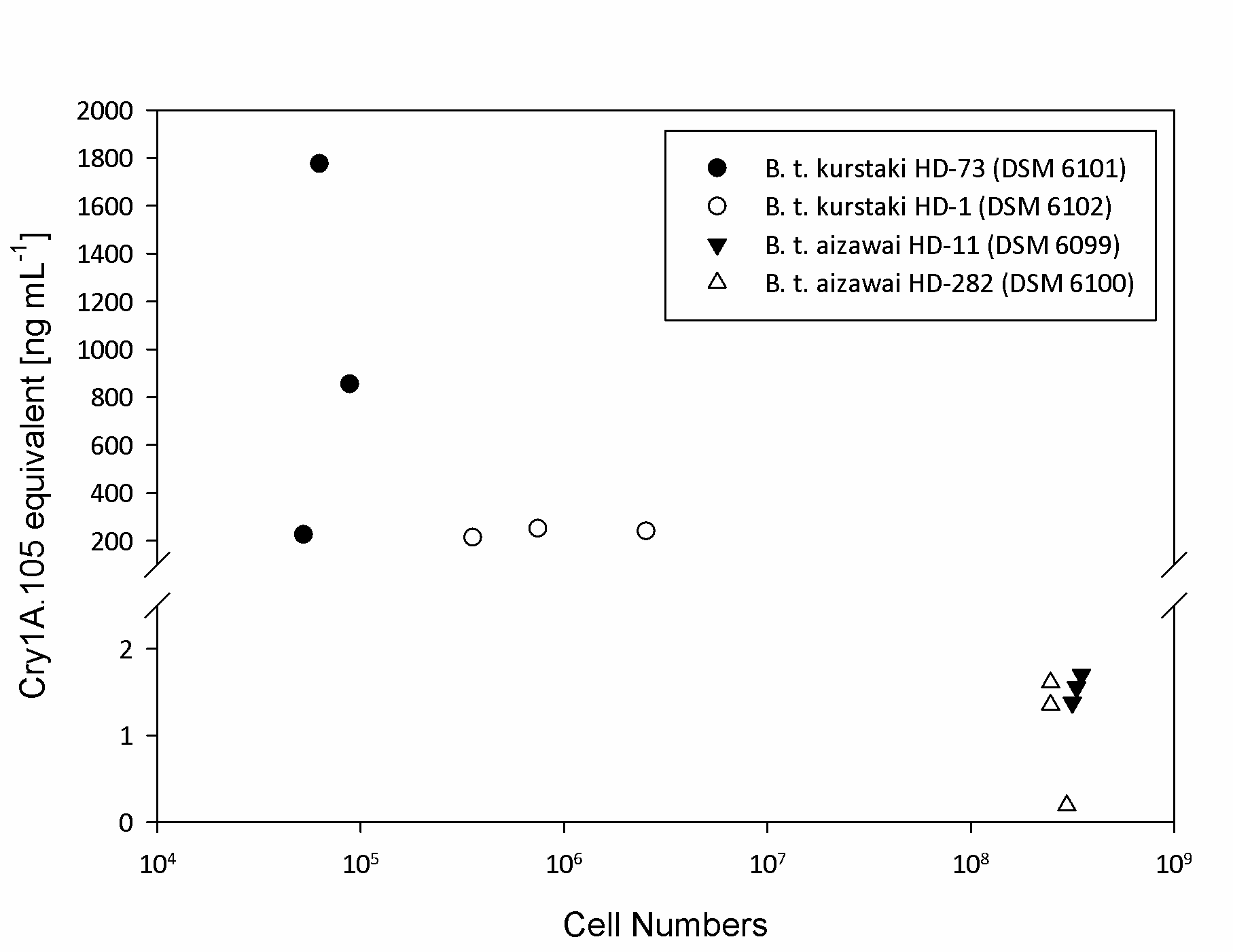

Supplement: Figure S2 — Quantification of natural Cry-protein expressed by four different Bacillus thuringiensis strains. The expression levels of natural Cry protein by four B. thuringiensis strains were detected with an ELISA targeting the synthetic protein Cry1A.105 as used in this study to detect the recombinant synthetic Cry1A.105 protein from Bt maize MON 89034 × MON 88017. The 12 data points are the highest diluted cell suspension with a signal above the respective detection limits. The results show for two type culture strains of ssp. kurstaki that a relatively low number of bacterial cells (spores) can result in detecting relative high amounts of Cry-protein. Contrastingly, the presence of a relative high numbers of the ssp. aizawai, show for two type culture strains, can result in detecting only low amounts of Cry-protein. No detection signal above the DTC was recorded for Bacillus subtilis 168 (DSM 402) (negative control). These results illustrate with the example of B. thuringiensis spp. kurstaki that Cry protein within the bee gut may originate from the presence of only a few bacterial cells (or spores). (TIF) [file pone.0059589.s002.tif]
