# Supplementary material for: Effect of Stacked Insecticidal Cry Proteins from Maize Pollen on Nurse Bees (Apis mellifera carnica) and Their Gut Bacteria
Source: PLoS One. 2013 Mar 22;8(3):e59589. doi: 10.1371/journal.pone.0059589 (PMC3606186; doi:10.1371/journal.pone.0059589)
Supplement: Table S1 — Consumption of Bt maize pollen by nurse bees within honey bee colonies. To indicate maize pollen exposure to bees, the pollen amount in midgut and hindgut samples was quantified by microscopic examination (Leitz Laboralux K, Wetzlar, Germany). By transferring each sample homogenate onto a counting device, complete pollen grains and fragments larger than half of a pollen grain were counted at 100×magnification within a 0.9 µL volume, at an 1∶4 dilution (Neubauer Improved haemocytometer, Laboroptik GmbH, Bad Homburg, Germany). Each count with four subsamples attributed with a factor 333 to the total number of pollen in the gut segment (0.9 µL/300 µL total sample volume). The counted pollen in the rectum samples (1305) indicated the presence of a total of 434,565 Bt-maize pollen; with an average exposure of 15,520 Bt-pollen per bee (n = 28), ±85.7% SD. Midgut samples did not contribute to additional exposure data of the Bt-maize pollen, because no pollen was observed (64 negative counts, in a total of 16 bees). The experimental colonies were free of pollen stores, and the nurse bees were at time of introduction less than 24 hrs old. As a result, no other pollen than maize pollen were found in the nurse bees from the bee cages. (DOCX) [file pone.0059589.s003.docx]

| Nurse ID | Sample 1 | Sample 2 | Sample 3 | Sample 4 | Total | Times 333 |
| --- | --- | --- | --- | --- | --- | --- |
| Bt45R | 49 | 51 | 39 | 29 | 168 | 55944 |
| Bt41R | 28 | 27 | 29 | 29 | 113 | 37629 |
| Bt49R | 21 | 24 | 26 | 24 | 95 | 31635 |
| Bt17R | 28 | 27 | 23 | 15 | 93 | 30969 |
| Bt27R | 25 | 22 | 21 | 22 | 90 | 29970 |
| Bt47R | 25 | 23 | 16 | 18 | 82 | 27306 |
| Bt53R | 19 | 22 | 24 | 15 | 80 | 26640 |
| Bt25R | 21 | 11 | 14 | 19 | 65 | 21645 |
| Bt43R | 14 | 14 | 15 | 18 | 61 | 20313 |
| Bt55R | 15 | 12 | 15 | 17 | 59 | 19647 |
| Bt03R | 13 | 9 | 12 | 16 | 50 | 16650 |
| Bt07R | 16 | 17 | 5 | 10 | 48 | 15984 |
| Bt13R | 11 | 9 | 12 | 9 | 41 | 13653 |
| Bt51R | 8 | 9 | 5 | 11 | 33 | 10989 |
| Bt31R | 12 | 6 | 6 | 7 | 31 | 10323 |
| Bt37R | 5 | 10 | 5 | 7 | 27 | 8991 |
| Bt21R | 6 | 4 | 10 | 6 | 26 | 8658 |
| Bt23R | 8 | 7 | 5 | 5 | 25 | 8325 |
| Bt09R | 5 | 8 | 8 | 2 | 23 | 7659 |
| Bt35R | 4 | 4 | 3 | 10 | 21 | 6993 |
| Bt29R | 6 | 3 | 8 | 2 | 19 | 6327 |
| Bt11R | 2 | 8 | 4 | 4 | 18 | 5994 |
| Bt01R | 2 | 5 | 3 | 3 | 13 | 4329 |
| Bt15R | 4 | 4 | 2 | 1 | 11 | 3663 |
| Bt19R | 1 | 1 | 3 | 3 | 8 | 2664 |
| Bt05R | 0 | 3 | 1 | 0 | 4 | 1332 |
| Bt33R | 0 | 0 | 0 | 1 | 1 | 333 |
| Bt39R | 0 | 0 | 0 | 0 | 0 | 0 |
