# Supplementary material for: Effect of Stacked Insecticidal Cry Proteins from Maize Pollen on Nurse Bees (Apis mellifera carnica) and Their Gut Bacteria
Source: PLoS One. 2013 Mar 22;8(3):e59589. doi: 10.1371/journal.pone.0059589 (PMC3606186; doi:10.1371/journal.pone.0059589)
Supplement: Table S2 — Comparison of 16S rRNA gene sequences retrieved in this study from the gut material of Apis mellifera and their affiliation to known bacteria taxa and previously detected bacterial 16S rRNA gene signatures in other studies. For the corresponding terminal restriction fragment (TRF) sizes, please see also Fig. 5. (DOCX) [file pone.0059589.s004.docx]

| T-RF (bp) in T-RFLP profiles^*^ | T-RF^**^ (bp) | Accession No. | Closest relative (Gene bank accession no.) | Similarity (%) | **Taxonomic classification^***^** | Group^****^ | Variety | Midgut | Hindgut | No. of clones |
| --- | --- | --- | --- | --- | --- | --- | --- | --- | --- | --- |
| ***Actinobacteria*** | | | | | | | | | | |
| 65 | 66 | HE613272 | Uncultured *Bifidobacterium* sp. (HM113200)^1^ | 99 | ***Bifidobacteriales*** | “Bifido” | Phacelia |  | x | 1 |
|  |  | HE613273-77 | Uncultured *Bifidobacterium* sp. (HM113220)^1^ | 96-99 |  | “Bifido” | DKC 5143 |  | x | 5 (1^*****^) |
| 161/164 | 165 | HE613278 | Uncultured *Bifidobacterium* sp. (HM113243)^1^ | 99 |  | “Bifido” | Phacelia |  | x | 1 |
|  |  | HE613279 | Uncultured *Bifidobacterium* sp. (HM113099)^1^ | 99 |  | “Bifido” | Benicia |  | x | 1 |
| ***Firmicutes*** | | | | | | | | | | |
| 318-320 | 322 | HE613280 | Uncultured bacterium (HM111911)^1^ | 99 | ***Bacilli*,**  ***Lactobacillales*** | F5 | Phacelia |  | x | 1 |
|  |  | HE613281-83 | Uncultured bacterium(HM113313)^1^ | 98-99 |  | F5 | Phacelia |  | x | 3 |
|  |  | HE613284-85 | Uncultured bacterium (HM113193)^1^ | 98 |  | F5 | DKC 5143 |  | x | 2 |
|  |  | HE613286-87 | Uncultured bacterium (HM111880)^1^ | 99 |  | F5 | DKC 5143 |  | x | 2 |
|  |  | HE613288 | Uncultured bacterium (HM113252)^1^ | 98 |  | F5 | DKC 5143 |  | x | 1 |
| ***Proteobacteria*** | | | | | | | | | | |
| 442 | 441 | HE613289-92 | Uncultured bacterium (HM112426)^2^ | 96 | ***Alphaproteobacteria*, *Acetobacteriacea*** | α-2 | BT | x |  | 4 (3^****^) |
| 449 | 447 | HE613293-97 | Uncultured bacterium (HM113219)^1^ | 98 | ***Betaproteobacteria*, *Neisseriaeceae*** | β-1 | BT | x |  | 5 (3^****^) |
|  | 448 | HE613298-99 | Uncultured bacterium (HM113170)^1^ | 98-99 |  | β-1 | Phacelia | x |  | 2 |
| 489 | 488 | HE613300 | Uncultured *Orbus* sp. (HM112036)^1^ | 98 | ***Gammaproteobacteria*, incertae sedis** | γ-1 | Benicia |  | x | 1 |
| 491/493 | 492 | HE613301 | Uncultured *Orbus* sp. (HM111973)^1^ | 98 |  | γ-1 | BT | x |  | 1 |
|  |  | HE613302 | Uncultured *Orbus* sp. (HM113151)^1^ | 98 |  | γ-1 | Phacelia | x |  | 1 |
|  |  | HE613303 | Uncultured *Orbus* sp. (HM112085)^1^ | 99 |  | γ-1 | Phacelia | x |  | 1 |
| ***Firmicutes*** | | | | | | | | | | |
| 568 | 567 | HE613304 | Uncultured bacterium (HM112042)^1^ | 99 | ***Bacilli*,  *Lactobacillales*, *Lactobacillaceae* (F4, F5)** | F4 | Phacelia |  | x | 1 |
|  | 569 | HE613305-06 | Uncultured bacterium (HM112126)^1^ | 98 |  | F5 | Phacelia |  | x | 2 (1^****^) |
| 570 | 570 | HE613307 | Uncultured bacterium (HM113344)^1^ | 98 |  | F4 | Phacelia |  | x | 1 |
|  |  | HE613308-09 | Uncultured bacterium (HM113214)^1^ | 99 |  | F5 | Phacelia | x |  | 2 (1^****^) |
|  |  | HE613310 | Uncultured bacterium (HM113344)^1^ | 99 |  | F5 | Phacelia | x |  | 1 |
| 580 | 579 | HE613311 | Uncultured bacterium (HM112788)^3^ | 98 |  | F4 | Phacelia | x |  | 1 |

^*^see Figure 5; ^**^T-RF as determined by *in silico* analyses of cloned DNA sequences; ^***^according to RDP classifier (see Materials and Methods), ^****^according to [23, 27]. ^*****^PCR fragments were cloned in reverse orientation, so that the determination of T-RFs was not possible. The assignment of these clones was done according to DNA sequence similarities (>97 %) to clones with known terminal sequences. DNA-sequences of closest relatives originated from bees (Family: Apidae), index numbers indicate different hosts ^1^*Apis mellifera*, ^2^*Xylocapa californica*, or ^3^*Diadasia opuntiae* [25]
