# Supplementary material for: Effect of Stacked Insecticidal Cry Proteins from Maize Pollen on Nurse Bees (Apis mellifera carnica) and Their Gut Bacteria
Source: PLoS One. 2013 Mar 22;8(3):e59589. doi: 10.1371/journal.pone.0059589 (PMC3606186; doi:10.1371/journal.pone.0059589)
Supplement: Table S3 — Comparison of bacterial community composition (T-RFLP profiles) between treatments (pollen source, i.e. BT, DKC, BEN, PHA) for mid- and hindgut by one-way analysis of similarities (ANOSIM) with Bray-Curtis similarity. (DOCX) [file pone.0059589.s005.docx]

|  | Midgut | | | | Hindgut | | | |
| --- | --- | --- | --- | --- | --- | --- | --- | --- |
| Pollen source | **0.083 ***** | | | | **0.123 ***** | | | |
|  |  | BT | PHA | DKC |  | BT | PHA | DKC |
|  | PHA | 0.079 ** | 0 |  | PHA | 0.164 *** | 0 |  |
|  | DKC | 0.039 ^n.s.^ | 0.142 *** | 0 | DKC | 0.11 ** | 0.116 *** | 0 |
|  | BEN | 0.082 ** | 0.074 ** | 0.081* | BEN | 0.130 *** | 0.103 ** | 0.109 *** |
